# Direct deposition on pre-cooled grids: a new approach to sub-millisecond cryoEM grid preparation

**DOI:** 10.1107/S2059798326007126

**Published:** 2026-08-11

**Authors:** David P. Klebl, Robert W. Kay, Sean McMillan, Frank Sobott, Nikil Kapur, Stephen P. Muench

**Affiliations:** a School of Biomedical Sciences, Faculty of Biomedical Sciences and Astbury Centre for Structural and Molecular Biology University of Leeds Leeds United Kingdom; b School of Mechanical Engineering University of Leeds Leeds United Kingdom; c School of Molecular and Cellular Biology, Faculty of Biological Sciences and Astbury Centre for Structural and Molecular Biology University of Leeds Leeds United Kingdom; Boston University School of Medicine, USA

**Keywords:** cryoEM, time-resolved cryoEM, structural biology

## Abstract

There has been a rapid expansion in new approaches to sample preparation for single-particle cryoEM. One drive has been around speeding up the process both for the application of time-resolved studies and in outrunning some of the negative effects which may come from the sample interacting with the air–water interface, such as degradation and preferred orientation. Here, we set out to significantly improve the speed of grid preparation by removing the plunging stage and directly spraying onto pre-cooled grids which are situated in the cryogen, allowing us to produce grids where two samples are mixed and frozen on the grid in less than 1 ms. We have previously shown a preliminary proof of principle with this new approach and here we provide a detailed description of the approach taken and the methodology behind it. This approach allows us to move beyond the millisecond timescale to open up more systems to time-resolved EM and potentially improve samples.

## Abstract 2

We describe a new method to prepare grids for cryoEM on the sub-millisecond timescale.

## 1. Introduction

Sample preparation for cryo electron microscopy (cryoEM) can, for some systems, still be a significant hurdle to obtaining high-resolution data, with common problems including sample degradation, preferred orientation and modification (Weissenberger, Henderikx *et al.*, 2021 ▸; Hirst *et al.*, 2024 ▸). It is still unclear why some proteins are affected more than others, but residence time on the grid and time in the thin-film environment of an electron microscopy (EM) grid are likely to play a role (D’Imprima *et al.*, 2019 ▸; Klebl, Gravett *et al.*, 2020 ▸). There are approaches to alleviate interactions with the air–water interface, which can include the use of support films such as graphene, detergents/surfactants or sacrificial proteins (Chen *et al.*, 2019 ▸; Russo & Passmore, 2014 ▸; Abe *et al.*, 2024 ▸). It is interesting to note that laser flash melting of an EM grid can also alleviate preferred orientation (Straub *et al.*, 2025 ▸).

In addition to the potential benefits to sample preparation, the ability to deposit and freeze on an EM grid will provide the ability to perform time-resolved cryoEM on the microsecond timescale when using a rapid mixing approach. There are alternative approaches that can achieve these timescales, such as the use of caged compounds and laser trapping, which can answer a range of biological questions (Lorenz, 2024 ▸). However, in some instances the use of a mixing and spraying approach, for example when no caged substrate is available, provides an alternative route.

When producing samples for cryoEM with conventional methods, there is a sequence of distinct steps that must be considered. The first is sample application to the grid. This is performed manually in Dubochet’s blotting method, for example with the Vitrobot system or manual plungers. Other systems directly write on a grid and avoid the blotting step, such as the Chameleon, Vitrojet or cryowriter, allowing controlled deposition of the sample in a thin film (Levitz *et al.*, 2022 ▸; Rima *et al.*, 2022 ▸; Ravelli *et al.*, 2020 ▸). Alternatively, other approaches avoid the blotting step by spraying small droplets of the sample onto the grid, for example Shake-It-Off or the VitriFlex (Rubinstein *et al.*, 2019 ▸; Peele *et al.*, 2025 ▸); with some setups, especially those focused on time-resolved applications, spraying onto a grid can reduce the time for this step. The next step is sample thinning. For a blotting system this is performed with a stationary grid and can take between 1 and 10 s, while this time is much reduced for more automated systems. The third step is grid transfer and vitrification. The time for this step is usually dictated by the maximum speed of the mechanical movement; most common are linear plungers that reach speeds of up to a few m s^−1^ and bridge distances of a few centimetres. Sample application and vitrification are typically separated by a suitable distance to accommodate the large temperature difference (sample application around ambient temperature and vitrification at cryogen temperature). A more detailed overview of different sample-preparation techniques can be found in Hirst *et al.* (2024 ▸).

An elegant way to produce grids without the need for blotting has recently been published where the sample grid is first plunged into liquid ethane to pre-cool before being rapidly raised from the cryogen and the protein directly sprayed on the grid and frozen before being re-plunged into the cryogen (Gusach *et al.*, 2025 ▸). Alternatively, specimens have also been prepared through directly spraying droplets into liquid ethane for time-resolved applications (Cherepanov & Schwalbe, 2023 ▸).

In the present work, we discuss how we achieved the sub-millisecond mixing and freezing reported in Klebl *et al.* (2022 ▸). We provide an alternative method to further reduce the time of cryoEM sample preparation to better prevent interactions between the sample and the air–water interface and to allow faster time-resolved cryoEM. We eliminate the plunging (transfer) step and apply the sample directly to a grid situated in the cryogen. Directly writing into the cryogen effectively combines the sample-application, thinning and vitrification steps into one and we estimate that it reduces the time of sample preparation to less than 1 ms. This approach may open up possibilities for new types of cryoEM experiments.

## 2. Methods

**Figure 1:**
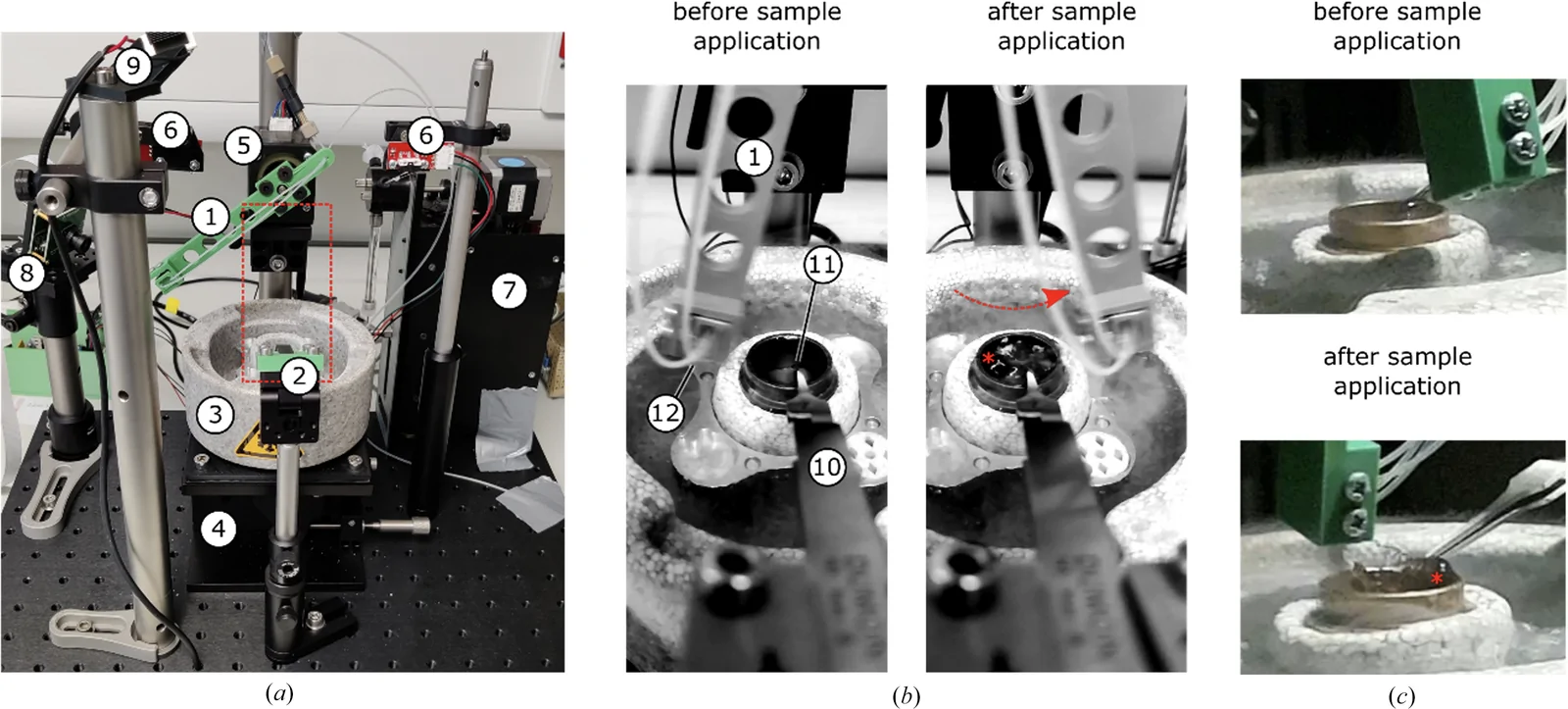
Experimental setup for plunge-free cryoEM grid preparation. Components are numbered as follows: 1, swinging arm; 2, grid holder; 3, nitrogen/ethane cup; 4, stage; 5, stepper motor; 6, arm limit switches; 7, syringe pump; 8, camera; 9, LED light source; 10, tweezers; 11, grid; 12, spray nozzle. (*a*) Overview of the device. The red dashed box indicates the region shown in (*b*). (*b*) Magnified front view showing the swinging arm, ethane cup and grid before and after sample application (left and right, respectively). The red dashed arrow (right) indicates the direction of movement of the arm and the red asterisk highlights the disturbance of the liquid ethane surface. (*c*) Magnified side view of the swinging arm before and after sample application (top and bottom, respectively). The red asterisk (bottom) highlights the disturbance of the liquid ethane surface.

A list of the main component parts for the experimental setup is given in Supplementary Table S1. The setup for plunge-free cryoEM grid preparation was built on an optical breadboard for accurate positioning of the components. A stepper motor (with the swinging arm), limit switches, tweezer holder, camera and LED were mounted on optical posts (Fig. 1 ▸*a*). Various holders and the swinging arm were 3D printed using an Ultimaker S5 with Ultimaker tough PLA filament. The limit switches, camera and LED were mounted on 25 or 12.7 mm optical posts. The height-adjustable stage for the ethane/nitrogen cup was fixed directly on the breadboard. The stepper motor was held by an L-bracket fixed on a 25 mm optical post, and with a damper between the bracket and motor. The swinging arm was mounted on the stepper motor shaft. The design of the arm was such that it holds a PDMS-based gas-dynamic virtual nozzle (Klebl, Monteiro *et al.*, 2020 ▸) at 10 cm distance from the motor shaft. Tweezers were held by a 3D-printed holder with a neodymium magnet on a 12.7 mm optical post, with adjustable height and angle.

Nitrogen gas supply to the nozzle was made as described for our time-resolved setup with appropriate connectors and sleeve tubing, with the gas pressure on the nozzle manually regulated (Klebl *et al.*, 2021 ▸). Commands to control liquid flow or valve position were sent to the syringe pump from the Raspberry Pi control computer via a USB/RS232 cable. The syringe pump was powered by a 24 V DC power supply.

The stepper motor and limit switches, responsible for moving/positioning the swinging arm, were connected to a RAMPS 1.4 shield on an Arduino Mega 2560 microcontroller. A DRV8825 motor driver was also mounted on the shield. Power to the shield and motor (12 V DC) was supplied by a 36 W power supply. The Arduino was programmed to move the motor counterclockwise until the swinging arm reaches the limit switch. Subsequently, the swinging arm would rotate the motor clockwise to move the arm back to the initial position. Motor speeds used were between 4 and 1.8 ms per step. A Python script with simple graphical user interface was used to control liquid flow and valve or plunger position, and to execute the run script. The graphical user interface was created with the *tkinter* module. Communication with the Arduino and syringe pump was perfomed using the *pyserial* module.

## 3. Results and discussion

### 3.1. General principle

To reduce the time of grid preparation, we investigated whether we could remove the third step of grid preparation, which was the slowest in our time-resolved cryoEM setup (Kontziampasis *et al.*, 2019 ▸), and whether it was possible to directly deposit sample onto a precooled grid. To expose a pre-cooled EM grid briefly to a sample spray, we used a swinging arm holding the same spray nozzle from our time-resolved setup that quickly passed over the grid. Conceptually, the approach is similar to aerosol jet printing in additive manufacturing, which is a contactless approach to directly write on a surface using a carrier gas to focus and deposit on a surface; this is reviewed in Wilkinson *et al.* (2019 ▸). An overview of the experimental setup is shown in Fig. 1 ▸. When passing over the grid, the gas flow through the spray nozzle briefly displaces the layer of ethane covering the grid, giving sufficient time for the sample to land on the grid (akin to parting of the waves) before the ethane returns (Figs. 1 ▸*b* and 1 ▸*c*). The exact point of freezing is yet to be determined but there may be residual liquid ethane on the grid, as seen in the approach described in Gusach *et al.* (2025 ▸), or at the point the ethane reflows over the surface of the grid. Based on the distance from the grid and the speed of droplets the ice should be formed in less than 1 ms. To investigate this, we performed a time-resolved experiment to mix ATP with the actomyosin complex, with the resultant mixed grids being consistent with sub-millisecond spraying and freezing; further details can be found in Klebl *et al.* (2022 ▸).

**Figure 2:**
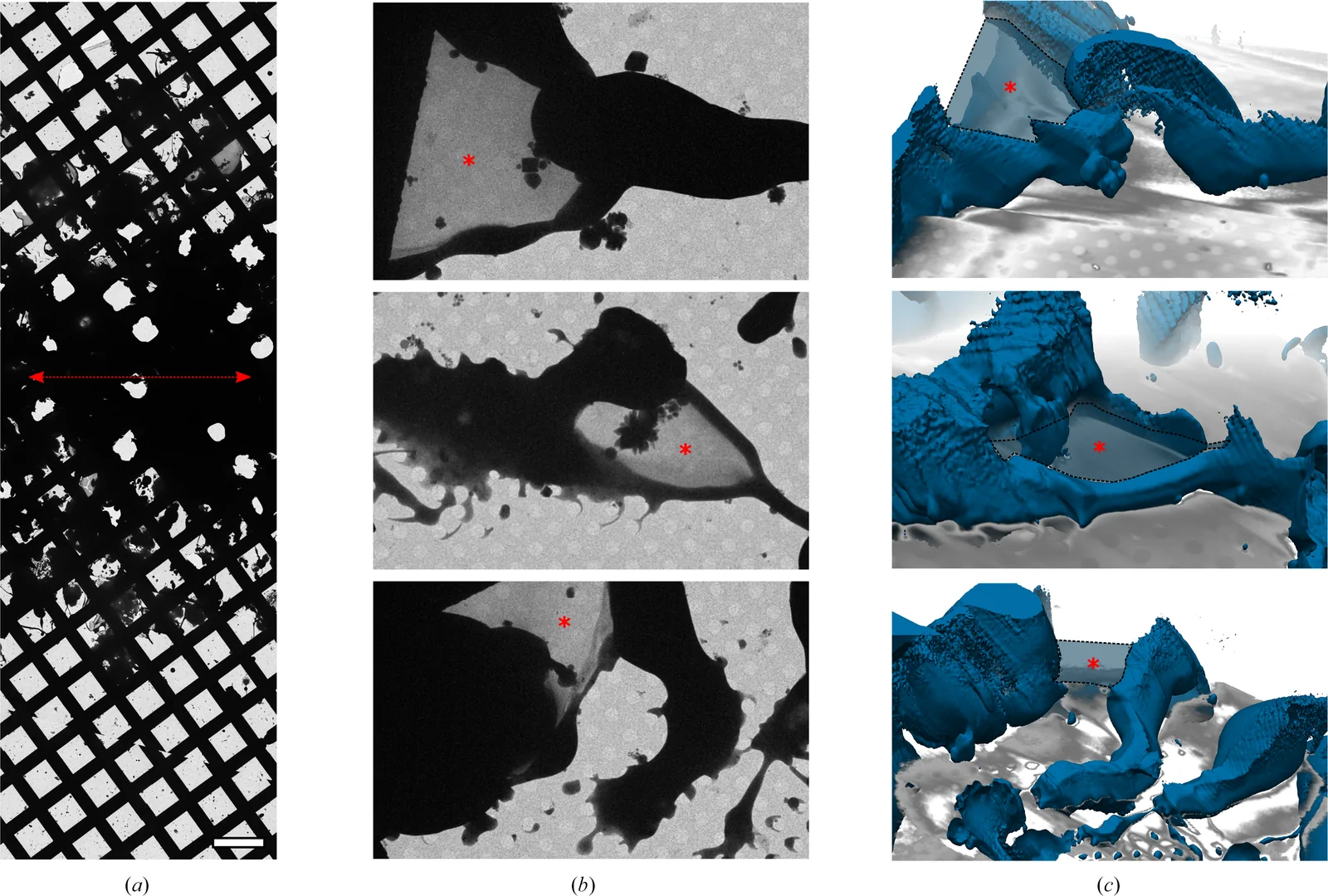
Low-magnification electron micrographs and tomographic reconstructions of the deposited thin sheets of vitreous ice. (*a*) Grid overview indicating the approximate direction of the spray nozzle with a dashed arrow. The scale bar corresponds to 100 µm. (*b*) Higher magnification images of the areas of ice suitable for imaging, highlighted with red asterisks. (*c*) Tomographic reconstructions of the areas shown in (*b*) with the grid surface as a grey slice and the supporting ice structure in dark blue, and indicating the position of the thin ice sheets in transparent blue (marked with red asterisks). Note that due to the large contour difference between the thick and thin areas the areas of thin ice are not visible at the contour level and are therefore represented by the area in transparent blue.

As expected, the sample is deposited as a diffuse stripe of droplets across the grid. At high liquid flow rates (>4 µl s^−1^, see below) the liquid in the centre of this line is too thick for cryoEM imaging (black grid squares in Fig. 2 ▸*a*). However, there are droplets towards the edge of the stripe that show areas of thin ice (Figs. 2 ▸*a* and 2 ▸*b*). The vitreous ice in these areas is sufficiently thin for cryoEM imaging. Acquisition of tilt series at low magnification revealed that these areas are sheets of thin ice, which are not in contact with the carbon film underneath but instead are supported by the surrounding rim of thick ice (Figs. 2 ▸*b* and 2 ▸*c*). The large variation in ice thickness across the grids makes reporting ‘typical’ ice difficult. Further work is required to better calculate the ice thickness through tomographic analysis over a large number of grid squares and repeats to produce significance in the values reported.

Next, we investigated the application of the new grid-preparation method for single-particle cryoEM and high-resolution structure determination (Klebl *et al.*, 2022 ▸). The results showed that ∼4.0 Å resolution was obtainable, albeit with a well behaved initial sample for proof of concept. We also explored the use of this rapid freezing approach for rapid mixing and spraying and its application for time-resolved studies, showing that we could obtain a time-resolution of sub-milliseconds (Klebl *et al.*, 2022 ▸). These are reported in our previous publication and will not be discussed in detail again here as we wish to focus on the experimental design. However, Table 1 ▸ shows the resolution of each structure in addition to the particle number and micrograph number to give a reflection on the modest data quality with this approach. The key parameters to be considered with the new approach reported here are listed in detail below.

### 3.2. Grid height and angle

We were lucky to find that our initial setup for grid height and angle was sufficient to obtain useable ice. Therefore, we did not systematically test the effect of the distance between the nozzle tip and grid, but currently find that a stand-off distance of 3–8 mm suffices to produce suitable ice. The grid is held under the cryogen using a standard set of Dumont N5 tweezers, typically at an angle of 30–45°. Grid tilt may not be strictly necessary to produce thin ice, with deposition onto grids held horizontally also possible, as discussed in Klebl *et al.* (2022 ▸). A further factor for consideration would be grid damage caused by the droplet spray and gas flow, with grid angle potentially playing a role in reducing the forces on the grid (by tilting) and reducing damage. The grids showed areas of damage (Fig. 2 ▸) in a position where the grid will have interacted with the sprayed droplets. Assessing the damage is more difficult in areas where thick ice exists. The current setup appears relatively robust; a systematic exploration of grid height and angle is likely to find a more optimal solution, but will also likely be influenced by other factors such as droplet and nozzle speed. Moreover, we have not yet investigated the effect of different cryogen temperatures/mixtures, for example the use of an ethane/propane mixture, and this may also provide an improvement in the ice quality seen and droplet behaviour.

### 3.3. Grid material

We have tested grids with copper mesh and a holey carbon foil, but have also used copper grids (hexagonal mesh) without foil, grids with an ultrathin carbon layer and self-wicking nanowire grids successfully. However, we did not see any noticeable difference in the subsequent ice quality. We hypothesize that this may be because the droplets have such a short residence time on the grid surface that the different geometries and materials do not have time to significantly influence this. Furthermore, as the grids are pre-cooled under the thin layer of liquid ethane the varying specific heats and masses of grids may not have a significant effect either, but further testing is required. The grid surface appears not to affect droplet spreading as it does with conventional plunging approaches, likely due to the short residence time on the grid before freezing. We also found that glow discharge of the grids is also not strictly necessary and the apparent effect of glow discharging may also be influenced by the very short residence time on the grid, limiting the time available for droplet spreading. Further work is required to fully investigate this, but the very short residence time on the grid will significantly reduce the influence of grid charging on droplet spreading. The knock-on effect of this is that it will reduce some of the ‘levers’ available to us to influence the speed of droplet spreading, with both grid type and glow discharging traditionally being important factors to consider when trying to obtain optimal ice.

### 3.4. Nozzle design and operation

A key factor for this new approach is providing a suitable flow of gas that will temporarily displace the liquid ethane from the grid surface, allowing the droplets to land and spread before freezing. Here, we used the same type of GDVN nozzle as described in Klebl, Monteiro *et al.* (2020 ▸), with a nitrogen gas pressure of 2–2.2 bar and a liquid flow rate of 4.2–16.7 µl s^−1^. Lower flow rates result in too few areas of thin ice. Based on previous work, we expect the resulting droplet speed to be ∼15–70 m s^−1^ with droplet sizes of 5–40 µm. Interestingly, our droplet speed is slower than that used by Gusach *et al.* (2025 ▸), and this highlights how sub-millisecond deposition and freezing can be achieved by a range of different approaches where nozzle geometries, droplet speed and gas flow can be optimized and refined. The spray gas serves a dual function: it is responsible for displacing the ethane and for atomizing the sample. Lower spray-gas pressures (0.5–1 bar), resulting in larger droplets, produced poorer quality grids and were not suitable.

### 3.5. Swinging-arm speed

The nozzle was moved over the grid at speeds of 0.8–1.7 m s^−1^. The physical relationship between the number of droplets ejected and the area that these cover suggests that significantly slower speeds will result in denser droplet deposition, and significantly faster speeds will require a higher liquid flow rate to achieve a comparable droplet density on the grid. We have not yet characterized what the limitations of this are and the range of speeds that are tolerable/optimal for the setup, but provide a starting point for others wishing to reproduce the methodology described here.

### 3.6. Current limitations and future directions

The apparatus described here moves away from the traditional plunging approach typical in other cryoEM sample-preparation methodologies and opens up new possibilities to be explored. It is clear from the resultant grids that significant optimization is required to increase the amount of useful ice on a single grid. Although we could collect sufficient data to solve a modest resolution structure, we would ideally require an order of magnitude more data to achieve those resolutions typically achieved (<3 Å). Other approaches such as the Chameleon and Vitrojet also only cover a limited portion of the grid, but the ice quality within these regions is significantly more consistent and thinner, allowing full data sets to be collected from just a few squares. The approach has proved to be relatively tolerant to a range of operating parameters, perhaps due to the gradient in ice thickness inherently capturing a region where successful imaging may be carried out. Nevertheless, it is likely that changes in deposition geometry, nozzle design and grid surface could be further optimized to increase the amount of useable area on the grid. Optimization of ice thickness and an increased yield of thin areas will be necessary to reach higher resolutions and make the method broadly applicable. Droplet spreading may be promoted by specific grid surfaces, depending on the relative timescales of inertial driven motion, surface-driven motion through wetting and phase change through freezing. The GDVN nozzle used for this study was not specifically designed for the new approach we report and the spray gas simultaneously acts to generate droplets and displace the ethane layer. There is clear scope for further improvements in droplet delivery, taking inspiration from other work which used aerosol-based 3D printing through a liquid layer. More advanced control could be implemented to reduce sample volume through enhanced synchronization of the spray, whilst recognizing the need for the spray to have properly established. It is interesting to note that despite the speeds of grid making available through this approach we did still see some association with the air–water interface for the ribosome, as discussed in Klebl *et al.* (2022 ▸). The morphology of the thin ice is similar to that seen in Gusach *et al.* (2025 ▸), and greater understanding of the mechanisms behind these approaches is key to unlocking the potential of these fast freezing approaches more widely.

Significant challenges still remain to be overcome for the new approach reported here to be more broadly taken up. The first lies in its optimization, as currently efficiency is low and not all grids have useable ice. Those that do typically have only a few squares of useable holes, which is tolerable for high-symmetry proteins where the particle number can be reduced, but will be a challenge for other systems. The other challenge is the low particle number, as also observed in Gusach *et al.* (2025 ▸). Solutions will be required to reduce the demand for protein concentrations typically 10 to 20 times higher than would be used in a blotting approach. Overcoming these challenges could unlock a significant change in what we can do in cryoEM. The first is in writing multiple samples on a cryoEM grid to improve the throughput, where grid changeover time is becoming a significant factor in data-collection time. The other advantage may be the improvement in particle quality. By reducing the amount of data discarded in image processing we can reduce the number of micrographs needed and computational cost of data processing.

## 4. Conclusions

Here, we report a method for rapidly depositing, thinning and vitrifying samples for cryoEM on the sub-millisecond timescale. The increase in speed for grid preparation and the decrease in the time the sample spends on the grid and in the thin-film environment could further mitigate some of the negative effects of the air–water interface on the sample and allows faster time-resolved cryoEM studies. Perhaps most importantly, it combines deposition, thinning and vitrification so that depositing multiple times on a single grid may become a reality with the approach reported here. Here, we provide a detailed description of the methodology and discuss some of the limitations and future potentials for the new approach. There is still the potential for much greater optimization of the approach and we hope that this work will drive new ideas within the community and allow new areas of science to be explored.

## Supplementary Material

Supplementary Table S1 and details of other supporting information. DOI: 10.1107/S2059798326007126/eh5026sup1.pdf

Supplementary Movie S1. DOI: 10.1107/S2059798326007126/eh5026sup2.mp4

.stl print file 1. DOI: 10.1107/S2059798326007126/eh5026sup3.bin

.stl print file 2. DOI: 10.1107/S2059798326007126/eh5026sup4.bin

.stl print file 3. DOI: 10.1107/S2059798326007126/eh5026sup5.bin

.stl print file 4. DOI: 10.1107/S2059798326007126/eh5026sup6.bin

.stl print file 5. DOI: 10.1107/S2059798326007126/eh5026sup7.bin

## Figures and Tables

**Table 1:** Overview of the resolution, micrograph number and particle number for the ribosome and apoferritin structures discussed More detailed analysis of these three samples was reported previously in Klebl *et al.* (2022 ▸).

Sample	Resolution (Å)	Particle No.	Micrograph No.
Ribosome (70S)	15	1123	110
Ribosome (50S)	12	2809	110
Ribosome (30S)	20	1803	110
Apoferritin	3.8	45482	511
