# Supplementary material for: Direct deposition on pre-cooled grids: a new approach to sub-millisecond cryoEM grid preparation
Source: Acta Crystallogr D Struct Biol. 2026 Aug 11;82(Pt 9):1056–61. doi: 10.1107/S2059798326007126 (PMC13532694; doi:10.1107/S2059798326007126)
Supplement: Supplementary file 1 [file d-82-01056-sup1.pdf]

STRUCTURAL BIOLOGY

Volume 82 (2026)

Supporting information for article:

Direct deposition on pre-cooled grids: a new approach to sub-millisecond cryoEM grid preparation

David K. Klebl, Robert W. Kay, Sean McMillan, Frank Sobott, Nikil Kapur and Stephen P. Muench

Table S1 Components of the new cryoEM grid-preparation setup.

**Mechanical components**	
Optical breadboard	Thorlabs MB3045/M - Aluminum Breadboard, 300 mm x 450 mm
	Thorlabs AV4/M - Ø27.0 mm Sorbothane Feet
Optical posts	Thorlabs TR300/M - Ø12.7 mm Optical Post
	2x Thorlabs TR150/M - Ø12.7 mm Optical Post
	2x Thorlabs RS300/M - Ø25.0 mm Pillar Post
	Thorlabs RS100/M - Ø25.0 mm Pillar Post
Optical post base/mounts	Thorlabs UPH150/M
	Thorlabs UPHA
	Thorlabs PH3T/M - Ø12.7 mm Translating Post Holder
	2x Thorlabs RS2P/M - Ø25.0 mm Pedestal Pillar Post
	2x Thorlabs CF125C/M - Clamping Fork
	Thorlabs CF175C/M – Clamping Fork
	Thorlabs RSH2/M Post Holder
Clamps and connectors	2x Thorlabs PMTR/M, for Ø1/2" Posts
	Thorlabs RA90RS/M Right-Angle Ø25.0 mm to Ø12.7 mm Post Clamp
Ethane/Nitrogen cup stage	TECHSPEC 125mm, Metric Z-Stage
Motor mount	Pololu Stamped Aluminum L-Bracket for NEMA 17 Stepper Motors
	Ooznest Stepper Motor Damper
	Ooznest Mounting Hub
Holders and platforms	Thorlabs C1015/M - Mounting Bracket for Ø25.0 mm Posts
	Thorlabs FP90/M – Adjustable Flip Platform
	Thorlabs C1019/M Adjustable-Height Platform for Ø25.0 mm Posts
**3D printed components**	
Holder for Ethane/Nitrogen container	-
Limit switch mount (2x)	-
Swinging arm	-
Tweezer holder	-
LED holder	-
Camera holder	-

**Electronics and computer control**	
Raspberry Pi	Raspberry Pi 4 Model B (4 GB RAM)
Arduino and USB cable	Arduino Board Mega 2560 (Rev3)
RAMPS shield for Arduino	RAMPS 1.4 Controller Board
Stepper motor driver	DRV8825 Stepper Motor Driver
Heatsink for DRV8825	Fischer Elektronik Heatsink, Universal Rectangular Alu, 74K/W, 8 x 8 x 6mm
NEMA17 stepper motor	Ooznest NEMA17 Stepper Motor (1.8° step)
Limit switch	2x WJMY mechanical limit switch for RAMPS 1.4
Syringe pump power supply	TODD SC24-10709 (24V, max. 8A)
USB to RS232 cable	BENFEI USB to RS-232/DB9(9-pin) Cable
Motor power supply	Ooznest 12V 36W DC Power Supply Adaptor
Raspberry Pi power supply	Raspberry Pi 4 Power Supply (5.1V, 3A)
Camera	Arducam OG02B10 2MP Colour
FPC cable for camera	Flex Cable for Raspberry Pi Camera or Display (610mm)
LED for illumination	Cree LED CXA2530
Power supply for LED	RS PRO LED Driver 45 W/1.05 A C.C. Output
**Other**	
Ethane container assembly	FEI Vitrobot ethane and nitrogen containers
Syringe pump	Kloehn Syringe Pump 50300 series (resolution 24,000 or 48,000) with 2-way valve
100 or 250 µL glass syringe	Kloehn
RS232 cable (to syringe pump)	Kloehn
1/8” steel tubing (for He)	e.g. IDEX (Cole-Parmer)
Liquid tubing and fittings	e.g. Upchurch (IDEX) or Cole-Parmer
Magnet for tweezer holder	Eclipse Block Magnet 5mm Neodymium, 0.74kg Pull
M6 and M4 screws	e.g. Thorlabs M4 & M6 hardware kits
Screw adapters	Thorlabs AE4M6M - Adapter with Internal M4 x 0.7 Threads and External M6 x 1.0 Threads
Tweezers	Dumont N7 type B

Supplementary Movie S1

A movie to show the grid placed under the liquid ethane with the spray device (attached to a green rotor arm) passing over and displacing the liquid ethane to deposit droplets.

Supplementary stl files

Corresponding .stl files for 3D printing the parts described within the paper for the camera holder, arm, end stop holder, ethane space, nozzle holder and tweezer holder.
